# Influence of *Lactobacillus plantarum* and *Pediococcus pentosaceus* addition on the nutritional quality of Sourdough-based bread

**DOI:** 10.1093/sumbio/qvaf030

**Published:** 2025-12-04

**Authors:** Amos Obaje Ogaji, Oluwatoyin Debby Coulthard, Michael Macvren Dashen, Tarfena Yoila Amapu, Isaac Amechi Onyimba, Deborah King Etah, Christiana Micah Umar, Samirah David Awak, Cirfat Nanya Amos, Anayochukwu Chibuike Ngene, Otumala John Egbere

**Affiliations:** Department of Microbiology, Faculty of Natural Sciences, University of Jos, P.M.B. 2084 Jos, Nigeria; Department of Microbiology, Faculty of Natural Sciences, University of Jos, P.M.B. 2084 Jos, Nigeria; Department of Microbiology, Faculty of Natural Sciences, University of Jos, P.M.B. 2084 Jos, Nigeria; Department of Science Laboratory Technology, Faculty of Natural Sciences, University of Jos, P.M.B. 2084 Jos, Nigeria; Department of Science Laboratory Technology, Faculty of Natural Sciences, University of Jos, P.M.B. 2084 Jos, Nigeria; Department of Microbiology, Faculty of Natural Sciences, University of Jos, P.M.B. 2084 Jos, Nigeria; Department of Microbiology, Faculty of Natural Sciences, University of Jos, P.M.B. 2084 Jos, Nigeria; Department of Microbiology, Faculty of Natural Sciences, University of Jos, P.M.B. 2084 Jos, Nigeria; Department of Microbiology, Faculty of Natural Sciences, University of Jos, P.M.B. 2084 Jos, Nigeria; Department of Microbiology, College of Natural Sciences, Michael Okpara University of Agriculture, Umudike, P.M.B. 7267 Umuahia, Nigeria; Department of Microbiology, Faculty of Natural Sciences, University of Jos, P.M.B. 2084 Jos, Nigeria

**Keywords:** sourdough fermentation, *Lactobacillus plantarum*, *Pediococcus pentosaceus*, nutritional enhancement, functional bread

## Abstract

This study explored the nutritional benefits of using a mixed culture of *Lactobacillus plantarum* and *Pediococcus pentosaceus* in sourdough bread fermentation. Over a 120-h fermentation period, spontaneous microbial activity resulted in peak lactic acid bacteria populations of 516.29 × 10^6^ cfu/g at 72 h in acha-based dough and 1082.78 × 10^6^ cfu/g at 24 h in rice-based dough (*P* < .001). The fermentation process led to a steady reduction in pH alongside a rise in titratable acidity. Bread produced with the mixed LAB cultures showed a significant decrease in phytic acid content and a notable enhancement in essential amino acids, such as lysine, leucine, isoleucine, valine, methionine, phenylalanine, and tryptophan, compared to the control. Furthermore, the energy value of the mixed culture bread (322.38 ± 0.63 Cal) was considerably higher than that of the control sample (266.73 ± 2.58 Cal). Overall, the incorporation of *L. plantarum* and *P. pentosaceus* during sourdough fermentation significantly boosted the nutritional quality of the bread, offering a valuable strategy for creating functional, nutrient-dense bakery products.

**Sustainability Statement**

This research directly supports Sustainable Development Goal 2 (Zero Hunger) by promoting the development of nutritionally enhanced sourdough bread using mixed cultures of *Lactobacillus plantarum* and *Pediococcus pentosaceus*. The improved amino acid profile, increased metabolizable energy, and reduced phytic acid content contribute to better dietary quality, particularly in regions where micronutrient deficiencies are prevalent. Additionally, the study aligns with SDG 3 (Good Health and Well-being) by offering a natural, functional food product that supports human health. Through the application of microbial biotechnology in food processing, this work also fosters sustainable innovation in the food industry in line with SDG 9 (Industry, Innovation, and Infrastructure).

## Introduction

Sourdough fermentation is an ancient biotechnology that has been used for centuries to improve the quality, nutritional value, and shelf life of bread (Huang et al. 2024, Onyimba et al. 2025). The fermentation process involves a complex microbial consortium of lactic acid bacteria (LAB) and yeasts, which contribute to the unique flavor, texture, and nutritional profile of sourdough bread (Corsetti and Settanni 2007, Arendt et al. 2007, Ohaegbu et al. 2025). Among the LAB, *Lactobacillus plantarum* and *Pediococcus pentosaceus* are commonly found in sourdough and are known for their ability to produce lactic acid, acetic acid, and other metabolites that enhance the nutritional and sensory properties of bread (De Vuyst and Neysens 2005, Arora et al. 2021, Egbere et al. 2021).

Recent studies have highlighted the potential of using mixed cultures of LAB in sourdough fermentation to further improve the nutritional quality of bread. Mixed cultures can enhance the proteolytic activity, reduce antinutritional factors such as phytic acid, and increase the bioavailability of essential amino acids (Limbad et al. 2020, Alkay et al. 2024). However, there is limited research on the effects of mixed cultures of *L. plantarum* and *P. pentosaceus* isolated from sourdough of Nigerian origin on the nutritional quality of sourdough bread.

This study aimed to investigate the effect of mixed cultures of *L. plantarum* and *P. pentosaceus* on the nutritional quality (proximate composition, amino acid profile, and antinutritional factors of sourdough bread). The findings of this study will contribute to the growing body of knowledge on the use of mixed LAB cultures in sourdough fermentation and provide insights into the development of nutritionally enhanced bakery products.

## Materials and methods

### Sample collection

Three brands of none-wheat flours (acha, rice, and sorghum) were purchased from Farin Gada Market Jos, transported in clean sterile rubber buckets to the Department of Microbiology, University of Jos for this study.

### Preparation of dough

Nonwheat flours (acha, rice, and sorghum) were subjected to spontaneous fermentation to produce sourdough. The flours were mixed with water (1:1 ratio) and allowed to ferment at 30°C for 120 h. The microbial counts, pH, and titratable acidity (TTA) were monitored at regular intervals.

### Microbial counts

Ten grams of each dough samples were homogenized in 90 ml of sterile peptone water. This was serially diluted up to 10^−6^ and the last two dilutions (10^5^ and 10^6^) were inoculated on de Man Rogosa Sharpes (MRS) agar for lactic acid bacteria count (LABC), plate count agar, plates for APC on potato dextrose agar (containing 0.5 mg/l streptomycin sulphate) for fungal count (FC). All inoculations were done in duplicate using the pour plate method. All MRS plates were incubated aerobically at 35°C for 24 h, while PDA plates were incubated at room temperature for 24 h. In all cases, colonies were counted after the incubation periods and results expressed as CFU/g (Zugic-Petrovic 2009, Dashen et al. 2020).

#### Purpose of microbial enumeration

Microbial counts were conducted to monitor the dynamic changes in microbial populations during the spontaneous fermentation of acha, rice, and sorghum flours. This analysis enabled the identification of the predominant LAB involved in sourdough development and the determination of their peak growth period. These data were used to select representative LAB strains (*L. plantarum* and *P. pentosaceus*) and establish the most active fermentation window for subsequent inoculation and bread production. Therefore, the microbial counts provided critical baseline information linking the natural sourdough microbiota to the nutritional improvements observed in the mixed-culture bread.

### Determination of pH of sourdough

Ten grams of each sourdough sample were blended with 90 ml of sterile distilled water, and the pH was measured using a pH meter, following the procedure described by Teixeira et al. (2024).

### Determination of TTA of sourdough

Ten millilitres of the homogenate from each sourdough samples were titrated against 0.1 N sodium hydroxide (NaOH) for the determination of TTA. The TTA was calculated using the formula below:

Percentage (%) TTA calculated as lactic acid:

TTA = Titre × Normality of base × chemical factor (0.009018) × 100 Weight of sample

(Wakil et al. 2014, Ohaegbu et al. 2022)

### Identification and characterization of LAB

The *L. plantarum* and *P. pentosaceus* strains used in this study were isolated from spontaneously fermented sourdough samples prepared from acha, rice, and sorghum flours as previously described. The isolates were purified and maintained on MRS agar slants at 4°C and as glycerol stocks (MRS broth supplemented with 10% glycerol) at −20°C. Identification was based on Gram staining, catalase reaction, and carbohydrate fermentation profiles using API 50 CHL kits, with confirmation against previously characterized LAB collections in the Department of Microbiology, University of Jos, Nigeria (Baratto et al. 2012, Ngene et al. 2023).

### Preparation of bread samples

Control breads and handling: For a valid comparison, control breads were prepared and handled under the same conditions as the experimental (mixed-culture) breads except for the addition of the test LAB cultures. The conventional control bread used the same formulation (1000 g flour, 640 ml tap water, 44 g salt, 40 g sugar) and the same manual mixing, leavening, proofing, and baking procedures described above, but without the addition of *L. plantarum* and *P. pentosaceus*. The conventional control dough received only 2 g compressed baker’s yeast. Doughs for both mixed-culture and conventional-control treatments were fermented at 30°C for 5 h, divided into 100 g dough balls, final-proofed at 35°C for 45 min and baked at 232°C for 25 min.

Two commercially produced bread samples (Commercial bread I and Commercial bread II) were included as additional external controls; these were purchased fresh from local bakeries in Jos on the day of analysis and handled identically during transport and sampling. All bread samples (mixed-culture, conventional-control, and commercial controls) were cooled to ambient temperature for 1 h after baking, packaged in sterile polyethylene bags, and analyzed within 24 h for proximate composition, antinutritional factors and amino acid profile. Each treatment was prepared and analyzed in triplicate (Rehman et al. 2007, Dashen et al. 2020).

#### Aseptic handling and replication

All utensils and pans were cleaned and sanitized before use. Samples were handled with sterile gloves and instruments during sampling and packaging to minimize cross-contamination. Each experimental treatment (mixed-culture and conventional-control) was prepared in three independent replicates and analysed in triplicate to allow statistical comparisons by ANOVA and DMRT.

### Nutritional analysis of baked bread

The proximate composition (moisture, crude protein, crude fiber, lipids, ash, and nitrogen-free extract) of the bread samples was determined using standard methods (AOAC 2019). The amino acid profile was analyzed using high-performance liquid chromatography (HPLC) (Maier et al. 2000). Phytic acid content was determined using the colorimetric method (Omotoso 2005).

### Statistical analysis

Data were analyzed using ANOVA, and mean values were separated using Duncan’s Multiple Range Test (DMRT).

## Results

### Microbial counts during the spontaneous fermentation of ‘‘acha’’ flour

Table 1 shows microbial counts during the spontaneous fermentation of ‘‘acha’’ flour. LAB were the most dominant population with the highest total mean count of 24.55 ± 1.05 × 10^7^ CFU/g, followed by a total mean FC of 20.02 ± 1.04 × 10^6^ CFU/g and the least was aerobic plate count (APC) of 2.42 ± 0.08 × 10^6^ CFU/g. There was a significant difference in the counts (*P* < .001). There was also a significant difference in the mean LABC, FC, and APC at the different hours of fermentation (*P* < .001).

**Table 1.** Microbial counts during spontaneous fermentation of ‘‘acha’’ flour

Fermentation time (H)	Mean ± SEM			ANOVA	*P*-value
	APC (CFU/g) (×10^6^)	LABC (CFU/g) (×10^7^)	FC (CFU/g) (×10^6^)		
0	$2.54\ \pm \ 0.02_{\mathrm{b}}^0$	$1.50\ \pm \ 0.50_{\mathrm{b}}^{\mathrm{c}}$	$19.00\ \pm \ 1.00_{\mathrm{a}}^{{\mathrm{cd}}}$	231.325	.001**
24	$2.59\ \pm \ 0.02_{\mathrm{b}}^0$	$3.50\ \pm \ 0.50_{\mathrm{b}}^{\mathrm{c}}$	$20.40\ \pm \ 0.60_{\mathrm{a}}^{{\mathrm{bc}}}$	494.753	<.001**
48	$2.68\ \pm \ 0.03_{\mathrm{c}}^0$	$6.90\ \pm \ 0.90_{\mathrm{b}}^{\mathrm{c}}$	$22.70\ \pm \ 1.10_{\mathrm{a}}^{\mathrm{b}}$	165.417	.001**
72	$2.21\ \pm \ 0.09_{\mathrm{c}}^0$	$9.75\ \pm \ 0.75_{\mathrm{b}}^{\mathrm{c}}$	$25.50\ \pm \ 0.50_{\mathrm{a}}^{\mathrm{a}}$	516.292	<.001**
96	$2.06\ \pm \ 0.01_{\mathrm{c}}^o$	$21.40\ \pm \ 0.60_{\mathrm{a}}^{\mathrm{b}}$	$17.00\ \pm \ 0.50_{\mathrm{b}}^{{\mathrm{de}}}$	505.326	<.001**
120	$2.47\ \pm \ 0.43_{\mathrm{c}}^o$	$24.55\ \pm \ 1.05_{\mathrm{a}}^{\mathrm{a}}$	$15.50\ \pm \ 0.10_{\mathrm{a}}^{\mathrm{e}}$	284.881	<.001**
Total mean	$2.42\ \pm \ 0.08_{\mathrm{c}}^0$	$11.27\ \pm \ 2.64_{\mathrm{b}}^o$	$20.02\ \pm \ 1.04_{\mathrm{a}}^a$	28.854	<.001**
AVONA	1.741	164.153	26.473		
*P*-value	0.259	< 0.001**	0.001**		

* Statistically significant difference exists at *P* ≤ .05.

** Statistically significant difference exists at *P* ≤ .01.

Values in the table are means of replicate readings. **Vertical comparison:** Mean values with different superscripts in the same column are significantly different. **Horizontal comparison:** Mean values with different subscripts in the same row are significantly different. Mean values were separated using DMRT.

### Microbial counts during the spontaneous fermentation of rice flour

The microbial counts during the spontaneous fermentation of rice are shown in Table 2. LAB had the highest total mean count of 28.40 ± 0.60 × 10^6^CFU/g, followed by a total mean APC of 8.70 ± 7.30 × 10^6^CFU/g, and the least was FC of 1.87 ± 0.17 × 10^6^CFU/g. There was a significant difference in the counts (*P* < .001). There was also a significant difference in the mean LABC, FC, and APC at the different hours of fermentation (*P* < .001).

**Table 2.** Microbial counts during spontaneous fermentation of rice flour

Fermentation time (H)	Mean ± SEM			ANOVA	*P*-value
	APC (CFU/g) (×10^6^)	LABC (CFU/g) (×10^6^)	FC (CFU/g) (×10^6^)		
0	$2.85\ \pm \ {{0.35}_{\mathrm{a}}}$	$0.32\ \pm \ 0.03_{\mathrm{b}}^{\mathrm{c}}$	$1.00\ \pm \ 0.02_{\mathrm{b}}^{\mathrm{b}}$	421.647	<.001**
24	$10.75\ \pm \ {{0.25}_{\mathrm{a}}}$	$0.68\ \pm \ 0.08_{\mathrm{c}}^{\mathrm{c}}$	$1.76\ \pm \ 0.20_{\mathrm{b}}^{\mathrm{b}}$	864.729	<.001**
48	$15.05\ \pm \ {{0.55}_{\mathrm{a}}}$	$1.15\ \pm \ 0.07_{\mathrm{b}}^{\mathrm{c}}$	$2.06\ \pm \ 0.03_{\mathrm{b}}^{\mathrm{b}}$	589.074	<.001**
72	$20.15\ \pm \ {{1.15}_{\mathrm{b}}}$	$1.67\ \pm \ 0.11_{\mathrm{c}}^{\mathrm{c}}$	$28.35\ \pm \ 1.65_{\mathrm{a}}^{\mathrm{a}}$	138.100	.001**
96	$17.10\ \pm \ {{0.40}_{\mathrm{b}}}$	$23.75\ \pm \ 0.75_{\mathrm{a}}^{\mathrm{b}}$	$2.02\ \pm \ 0.05_{\mathrm{c}}^{\mathrm{b}}$	512.981	<.001**
120	$8.70\ \pm \ {{7.30}_{\mathrm{b}}}$	$28.40\ \pm \ 0.60_{\mathrm{a}}^{\mathrm{a}}$	$1.87\ \pm \ 0.17_{\mathrm{b}}^{\mathrm{b}}$	10.609	.044*
Total mean	13.27 ± 1.64	9.33 ± 3.60	6.17 ± 3.00	1.539	.230
AVONA	2.645	1082.783	254.018		
*p*-value	0.134	< 0.001**	<0.001**		

* Statistically significant difference exists at *P* ≤ .05. **Statistically significant difference exists at *P* ≤ .01.

Values in the table are means of replicate readings. **Vertical comparison:** Mean values with different superscripts in the same column are significantly different. **Horizontal comparison:** Mean values with different subscripts in the same row are significantly different. Mean values were separated using DMRT.

### Microbial counts during the spontaneous fermentation of sorghum flour

The highest total mean count was LAB with a count of 9.76 ± 3.61 × 10^7^CFU/g, followed by total mean FC of 13.14 ± 2.75 × 10^6^CFU/g and the least was APC of 1.04 ± 0.22 × 10^6^CFU/g. The total mean counts differ significantly (*P* < .001). There was also a significant difference in the mean LABC, FC, and APC at the different hours of fermentation (*P* < .001) (Table 3).

**Table 3.** Microbial counts during spontaneous fermentation of sorghum flour

Fermentation time (H)	Mean ± SEM			ANOVA	*P*-value
	APC (CFU/g) (×10^6^)	LABC (CFU/g) (×10^6^)	FC (CFU/g) (×10^6^)		
0	$0.92\ \pm \ 0.07_{\mathrm{a}}^{\mathrm{c}}$	$0.30\ \pm \ 0.05_{\mathrm{b}}^{\mathrm{b}}$	$0.06\ \pm \ 0.01_{\mathrm{c}}^{\mathrm{c}}$	78.026	.003**
24	$1.03\ \pm \ 0.09_0^{\mathrm{c}}$	$1.40\ \pm \ 0.20_0^{\mathrm{b}}$	$0.88\ \pm \ 0.03_{\mathrm{a}}^{\mathrm{c}}$	4.480	.126
48	$1.78\ \pm \ 0.11_{\mathrm{b}}^{\mathrm{b}}$	$1.77\ \pm \ 0.10_{\mathrm{b}}^{\mathrm{b}}$	$16.85\ \pm \ 0.15_{\mathrm{a}}^{\mathrm{b}}$	5211.030	<.001**
72	$2.11\ \pm \ 0.02_{\mathrm{b}}^{\mathrm{a}}$	$2.18\ \pm \ 0.13_{\mathrm{b}}^{\mathrm{b}}$	$21.70\ \pm \ 1.70_{\mathrm{a}}^{\mathrm{a}}$	131.623	.001**
96	$0.27\ \pm \ 0.01_{\mathrm{b}}^{\mathrm{d}}$	$24.75\ \pm \ 4.05_{\mathrm{a}}^{\mathrm{a}}$	$19.65\ \pm \ 0.95_{\mathrm{a}}^{{\mathrm{ab}}}$	28.923	.011*
120	$0.13\ \pm \ 0.03_{\mathrm{c}}^{\mathrm{d}}$	$28.15\ \pm \ 0.65_{\mathrm{a}}^{\mathrm{a}}$	$19.70\ \pm \ 0.70_{\mathrm{b}}^{{\mathrm{ab}}}$	678.490	<.001**
Total mean	$1.04\ \pm \ {{0.22}_{\mathrm{b}}}$	$9.76\ \pm \ {{3.61}_{\mathrm{a}}}$	$13.14\ \pm \ {{2.75}_{\mathrm{a}}}$	5.666	.008**
AVONA	145.900	59.942	137.636		
*P*-value	<.001**	<.001**	<.001**		

* Statistically significant difference exists at *P* ≤ .05. **Statistically significant difference exists at *P* ≤ .01

Values in the table are means of replicate readings. **Vertical comparison:** Mean values with different superscripts in the same column are significantly different. **Horizontal comparison:** Mean values with different subscripts in the same row are significantly different. Mean values were separated using DMRT.

### Comparison of the total mean microbial counts of “acha,” rice, and sorghum flours during spontaneous fermentation

Table 4 shows the comparison of the total mean counts of the three different flours. “Acha” had the highest total mean LABC of 11.27 ± 2.64 × 10^7^CFU/g, followed by rice with a total mean LABC of 9.33 ± 3.60 × 10^6^CFU/g and sorghum 11.27 ± 2.64 × 10^6^CFU/g. There is a significant difference in the counts (*P* < .001). Acha also had the highest total mean FC of 20.02 ± 1.04 × 10^6^CFU/g, while rice had the highest total mean APC of 13.27 ± 1.64 × 10^6^CFU/g.

**Table 4.** Comparison of the total mean microbial counts of acha, rice and sorghum flours during spontaneous fermentation

Flour	LABC (CFU/g)	FC (CFU/g)	APC	ANOVA	*P-*value
Acha	11.27 ± 2.64 × 10^7a^	20.02 ± 1.04 × 10^6a^	2.42 ± 0.08 × 10^6a^	28.854	<.001*
Rice	9.33 ± 3.60 × 10^6^	6.17 ± 3.00 × 10^6^	13.27 ± 1.64 × 10^6^	1.539	0.230
Sorghum	9.76 ± 3.61 × 10^6b^	13.14 ± 2.75 × 10^6c^	1.04 ± 0.22 × 10^6c^	5.666	<.008*

* Statistically significant difference exists at *P* ≤ .01. Values in the table are means of replicate readings. Mean values with different superscripts in the same row are significantly different. Mean values were separated using DMRT.

### pH and TTA of sourdough and bread

The pH of the sourdough samples decreased progressively during fermentation, while TTA increased correspondingly. The final pH values recorded were 3.90 ± 0.10 for *acha*, 4.20 ± 0.00 for rice, and 4.00 ± 0.00 for sorghum. Similarly, the final TTA values were 0.489 ± 0.013 for *acha*, 0.407 ± 0.002 for rice, and 0.489 ± 0.013 for sorghum. The mixed culture bread had the lowest pH and higher TTA of 5.25 ± 0.25^c^ and 0.492 ± 0.014^b^, respectively, compared with the control bread samples (Tables 5 and 6).

**Table 5.** pH and TTA during the spontaneous fermentation of the flours

	pH					TTA				
Time (h)	Acha	Rice	Sorghum	ANOVA	*P*-value	Acha	Rice	Sorghum	ANOVA	*P*-value
0	6.55 ± 0.05^a^	6.50 ± 0.00^a^	6.65 ± 0.15^a^	0.700	.563	0.163 ± 0.006^e^	0.160 ± 0.001^e^	0.160 ± 0.001^e^	0.246	.796
24	6.10 ± 0.10^b^	6.25 ± 0.25^ab^	6.60 ± 0.00^a^	2.724	.212	0.172 ± 0.005^e^	0.170 ± 0.007^e^	0.161 ± 0.005^e^	1.254	.402
48	5.80 ± 0.20^b^	5.80 ± 0.30^bc^	5.65 ± 0.05^b^	0.170	.851	0.224 ± 0.010^d^	0.210 ± 0.010^d^	0.216 ± 0.003^d^	0.704	.562
72	5.15 ± 0.15^c^	5.35 ± 0.15^c^	5.20 ± 0.10^c^	0.591	.608	0.273 ± 0.003^c^	0.275 ± 0.003^c^	0.281 ± 0.005^c^	1.232	.407
96	4.70 ± 0.10^d^	4.50 ± 0.00^d^	4.75 ± 0.15^d^	1.615	.334	0.357 ± 0.017^b^	0.378 ± 0.001^b^	0.355 ± 0.015^b^	0.997	.466
120	3.90 ± 0.10^e^	4.20 ± 0.00^d^	4.00 ± 0.00^e^	7.000	.074	$0.489\ \pm \ 0.013_{\mathrm{a}}^{\mathrm{a}}$	$0.407\ \pm \ 0.002_{\mathrm{b}}^{\mathrm{a}}$	$0.489\ \pm \ 0.013_{\mathrm{a}}^{\mathrm{a}}$	20.987	.017*
Total	5.37 ± 0.27	5.43 ± 0.26	5.48 ± 0.29	0.040	.961	0.279 ± 0.035	0.267 ± 0.029	0.277 ± 0.035	0.041	.960
**ANOVA**	60.232	29.749	113.817			160.617	439.444	229.011		
*P* **-value**	<.001**	<.001**	<.001**			<.001**	<.001**	<.001**		

* Statistically significant difference exists at *P* ≤ .05. **Statistically significant difference exists at *P* ≤ .01

Values in the table are means of replicating readings. **Vertical comparison:** Mean values with different superscripts in the same column are significantly different. **Horizontal comparison:** Mean values with different subscripts in the same row are significantly different. Mean values were separated using DMRT.

**Table 6.** pH and TTA of the bread samples

Sample	Mean ± SEM	
	pH	TTA
Mixed culture bread	5.25 ± 0.25^c^	0.492 ± 0.014^b^
Conventional bread (Control)	6.60 ± 0.00^a^	0.222 ± 0.019^d^
Commercial bread I (Control)	6.55 ± 0.25^ab^	0.215 ± 0.015^d^
Commercial bread II (Control)	6.60 ± 0.20^a^	0.227 ± 0.024^d^
**ANOVA**	4.925	32.535
***P*-value**	.010*	<.001**

### Proximate composition of the bread samples

The result of the proximate composition of the bread samples is shown in Table 7. The mixed culture bread had lower protein content of 10.27 ± 0.00 compared to the conventional bread (control), commercial bread I (control) and commercial bread II (control) with protein content of 10.57 ± 0.24, 12.95 ± 0.03, and 11.12 ± 0.15, respectively. However, the mixed culture bead had higher metabolizable energy of 322.38 ± 0.63 compared to metabolizable energy of 266.73 ± 2.58, 305.05 ± 0.45, and 319.83 ± 2.73 for conventional bread (control), commercial bread I (control) and commercial bread II (control) respectively. The differences in protein content and metabolizable energy are significant at *P* < .001.

**Table 7.** Proximate composition of the baked products

Sample	Mean ± SEM (%)						
	Moisture	Crude Protein	Crude Fibre	Lipids	Ash	Nitrogen Free Extract	Metabolizable Energy (Cal)
Mixed culture bread	25.13 ± 0.08^c^	10.27 ± 0.00^e^	5.30 ± 0.20^c^	9.78 ± 0.03^a^	1.20 ± 0.00	48.33 ± 0.10^d^	322.38 ± 0.63^b^
Conventional bread (Control)	29.35 ± 0.20^a^	10.57 ± 0.24^de^	5.50 ± 0.00^bc^	2.33 ± 0.03^f^	1.38 ± 0.48	50.89 ± 0.94^bc^	266.73 ± 2.58^e^
Commercial bread I (Control)	22.58 ± 0.48^de^	12.95 ± 0.03^a^	6.35 ± 0.05^a^	5.15 ± 0.15^e^	1.25 ± 0.35	51.73 ± 0.26^b^	305.05 ± 0.45^d^
Commercial bread II (Control)	22.88 ± 0.53^de^	11.12 ± 0.15^cd^	6.25 ± 0.05^a^	7.83 ± 0.13^c^	0.70 ± 0.05	51.23 ± 0.25^b^	319.83 ± 2.73^bc^
**ANOVA**	68.628	20.504	17.212	178.754	0.915	20.621	78.882
***P*-value**	<.001**	<.001**	<.001**	<.001**	.549	<.001**	<.001**

* Statistically significant difference exists at *P* ≤ .05. **Statistically significant difference exists at *P* ≤ .01

Values in the table are means of replicate readings. Mean values with different superscripts in the same column are significantly different. Mean values were separated using DMRT.

### Antinutritional factors of the bread samples

There was a considerable reduction in the phytic, tannin, and oxalate content of the mixed culture bread compared to the control bread. Mixed culture bread and commercial bread I had the least tannins and oxalate contents of 3.99 ± 0.10 mg and 8.00 ± 0.50 mg, respectively There was significant difference (*P* < .001) in the antinutritional content as shown in Table 8.

**Table 8.** Antinutritional factors of the bread samples

Sample	Mean ± SEM		
	Phytic acid (mg/100 g)	Tannins (mg/100 g)	Oxalate (mg/100 g)
Mixed culture bread	2.63 ± 0.13^d^	3.99 ± 0.10^f^	8.75 ± 1.25^de^
Conventional bread (Control)	13.01 ± 0.23^a^	7.33 ± 0.39^de^	36.68 ± 1.68^b^
Commercial bread I (Control)	9.59 ± 0.46^c^	15.59 ± 0.48^b^	19.00 ± 1.50^c^
Commercial bread II (Control)	11.04 ± 0.99^b^	21.12 ± 0.93^a^	8.00 ± 0.50^e^
**ANOVA**	120.387	67.211	177.557
***p*-value**	<.001**	<.001**	<.001**

* Statistically significant difference exists at *P* ≤ .05. **Statistically significant difference exists at *P* ≤ .01

Values in the table are means of replicate readings. Mean values with different superscripts in the same column are significantly different. Mean values were separated using DMRT.

### Amino acid profile of the bread samples

The amino acid content of mixed culture bread was significantly higher than that of control bread samples (*P* ≤ .001). The cysteine, lysine, tyrosine, isoleucine, tryptophan, phenylalanine, and content of the mixed culture bread increased by 17.88%, 15.84%, 15.27%, 12.59%, 10.99%, and 7.39%, respectively. There was also a 5.97%, 4.68%, 4.60%, and 4.35% increase in the valine, arginine, alanine, and glumatic acid content of the mixed culture bread, respectively, in comparison with the mean content of the three control bread samples (Tables 9 and 10).

**Table 9.** Amino acid profile of the bread samples

Sample	Mean ± SEM (g/100 g)						
	Leucine	Lysine	Isoleucine	Phenylalanine	Tryptophan	Valine	Methionine
Mixed culture bread	6.18 ± 0.02^bc^	3.41 ± 0.02^c^	4.05 ± 0.02^c^	4.33 ± 0.03^e^	1.31 ± 0.00^c^	4.52 ± 0.02^c^	1.22 ± 0.02^de^
Conventional bread (Control)	5.44 ± 0.03^d^	2.68 ± 0.01^f^	3.52 ± 0.02^de^	3.80 ± 0.02^h^	1.12 ± 0.02^e^	4.04 ± 0.03^e^	1.19 ± 0.02^e^
Commercial bread I (Control)	5.80 ± 0.11^cd^	3.10 ± 0.16^d^	3.88 ± 0.08^cd^	4.25 ± 0.01^e^	1.19 ± 0.03^d^	4.41 ± 0.06^cd^	1.31 ± 0.05^cd^
Commercial bread II (Control)	5.63 ± 0.03^cd^	2.83 ± 0.03^ef^	3.22 ± 0.52^e^	3.97 ± 0.03^g^	1.02 ± 0.00^f^	4.29 ± 0.02^d^	1.29 ± 0.01^d^
**ANOVA**	5.653	37.046	6.697	224.776	52.856	41.665	9.259
***p*-value**	0.006**	< 0.001**	0.003**	< 0.001**	< 0.001**	< 0.001**	0.001**

* Statistically significant difference exists at *P* ≤ .05. **Statistically significant difference exists at *P* ≤ .01

Values in the table are means of replicate readings. Mean values with different superscripts in the same column are significantly different. Mean values were separated using DMRT.

**Table 10.** Amino acid profile of the bread samples

Sample	Mean ± SEM (g/100 g)						
	Proline	Arginine	Tyrosine	Histidine	Cysteine	Alanine	Glutamic acid
Mixed culture bread	7.46 ± 0.05	4.27 ± 0.03^de^	2.73 ± 0.03^d^	2.45 ± 0.04^ef^	1.51 ± 0.01^d^	3.67 ± 0.03^cd^	16.55 ± 0.05^d^
Conventional bread (Control)	6.72 ± 0.02	4.22 ± 0.01^de^	2.26 ± 0.02^g^	2.35 ± 0.02^f^	1.08 ± 0.02^g^	3.52 ± 0.03^fg^	15.30 ± 0.09^g^
Commercial bread I (Control)	7.44 ± 0.13	4.16 ± 0.12^de^	2.59 ± 0.01^e^	2.57 ± 0.08^de^	1.39 ± 0.06^e^	3.57 ± 0.04^ef^	16.14 ± 0.05^ef^
Commercial bread II (Control)	7.05 ± 0.04	3.84 ± 0.03^f^	2.43 ± 0.02^f^	2.44 ± 0.01^ef^	1.26 ± 0.01^f^	3.45 ± 0.00^g^	16.05 ± 0.00^f^
**ANOVA**	1.030	54.116	288.171	48.828	319.754	99.824	450.201
***P*-value**	.478	<.001**	<.001**	<.001**	<.001**	<.001**	<.001**

* Statistically significant difference exists at *P* ≤ .05. **Statistically significant difference exists at *P* ≤ .01

Values in the table are means of replicate readings. Mean values with different superscripts in the same column are significantly different. Mean values were separated using DMRT.

## Discussion

The microbial enumeration provided insight into the fermentation dynamics of the cereal substrates, confirming LAB dominance and validating the selection of *L. plantarum* and *P. pentosaceus* as key functional strains for subsequent bread enrichment. The microbial succession during spontaneous fermentation of acha, rice, and sorghum flours demonstrated significant variations in bacterial and fungal dynamics over time, with LAB consistently showing progressive proliferation across all substrates, particularly in acha and sorghum where LABCs increased from initial low levels (1.50 × 10^7^ CFU/g and 0.30 × 10⁵ CFU/g, respectively) to peak values at 120 h (24.55 × 10^7^ CFU/g in acha and 28.15 × 10^6^ CFU/g in sorghum), while rice flour exhibited a similar LAB growth trend albeit starting from a lower baseline (0.32 × 10^6^ CFU/g to 28.40 × 10^6^ CFU/g), reflecting the critical role of LAB in spontaneous fermentation, a pattern corroborated by previous studies that highlight their metabolic adaptability to cereal substrates (Liu et al. 2023). APCs increased significantly in rice and sorghum from 0 to 72 h, reaching peaks of 20.15 × 10^6^ CFU/g in rice and 2.11 × 10^6^ CFU/g in sorghum before declining by 120 h. In contrast, acha flour showed less variation in APC (2.54 × 10^6^ to 2.47 × 10^6^ CFU/g), suggesting that acha supports a relatively stable bacterial load during fermentation, possibly due to its unique nutritional composition or indigenous microbiota (Adebiyi et al. 2016). FCs presented diverse patterns; in acha and sorghum, the fungal load increased significantly, peaking at 25.50 × 10^6^ CFU/g and 21.70 × 10^6^ CFU/g at 72 h, respectively, while rice flour exhibited a transient rise in fungal load at 72 h (28.35 × 10^6^ CFU/g) followed by a sharp decline, indicating potential substrate-specific selective pressures or microbial antagonism between fungi and LAB (Tamang et al. 2016). The statistically significant changes (*P* < .001) in LAB and FCs across fermentation times in all flours, confirmed by ANOVA and post-hoc analysis, emphasize the dynamic interplay between bacterial proliferation and fungal suppression or coexistence during natural fermentation, a microbial pattern consistent with the establishment of lactic acid fermentation dominance in cereal-based substrates (Corsetti and Settanni 2007).

The reduction in pH and increase in acidity were more pronounced in *acha* and sorghum sourdoughs, possibly due to the higher LAB population. Bread made with 20% sourdough had the lowest pH (5.25 ± 0.25) and the highest TTA (0.588 ± 0.018), indicating stronger acidification compared to commercial and control bread samples. Conversely, commercial bread I had the lowest TTA (0.215 ± 0.015), suggesting minimal microbial acidification.

The progressive decrease in pH and increase in TTA during the spontaneous fermentation of the flours into sourdough has been reported by other researchers in this area (Savic et al. 2007, Saeed et al. 2009, Dashen et al. 2017, Dashen et al. 2020). The decrease in pH and increase in TTA is due to lactic acid and acetic acid production from simple sugars by the LAB during sourdough fermentation (Chochkov et al. 2023). The final pH (3.90) obtained at the end of ‘‘acha’’ sourdough fermentation is similar to that reported by Dashen et al. (2020) and Chochkov et al. (2023) who reported pH of 3.80 and 3.84 as the final pH of wheat sourdough fermentation, respectively.

The decreased pH and increased TTA in bread prepared with the mixed culture result from the metabolic processes of LAB, which ferment available sugars into organic acids, including lactic acid, acetic acid, and carbon dioxide. This acid production lowers the dough’s pH and elevates TTA, aligning with findings from studies on sourdough fermentation (Park et al. 2007, Han et al. 2018, Katke et al. 2019, Woo et al. 2023).

The significantly higher metabolizable energy of the mixed culture bread compared to the control bread samples implies that LAB starter bread as a good source of dietary energy to consumers of sourdough bread.

The lower crude protein content of the mixed culture bread compared to the three-control bread may be explained by the fact that LAB contributes to overall proteolysis during dough fermentation by creating optimum conditions (acidification)for the activity of cereal proteases (Saeed et al. 2009, Dashen et al. 2017).

The use of mixed culture of LAB as starter cultures led to a significant decrease in the phytic acid content of the mixed culture bread. According to Limbad et al. (2020), the stimulation of endogenous grain phytase and the phytase activity of the LAB and yeast due to pH decrease during the sourdough fermentation is responsible for the decrease in phytate level in sourdough and LAB bread.

The findings from this study demonstrate that mixed culture sourdough fermentation using *L. plantarum* and *P. pentosaceus* significantly enhances the essential amino acid profile of bread, particularly increasing lysine, leucine, isoleucine, valine, phenylalanine, methionine, and tryptophan compared to conventional and commercial breads, though the enhancement is less pronounced in than in high-concentration sourdough or *L. plantarum* monoculture breads, which aligns with the reports of Rizzello et al. (2016) and Gobbetti et al. (2019).

The observed enhancements underscore the contribution of sourdough fermentation to improving bread’s nutritional profile, particularly by enhancing protein digestibility and enriching amino acid content. These results align with studies by Limbad et al. (2020), which noted elevated amino acid levels in *L. plantarum*-fermented bread. Alkay et al. (2024) suggest that acidification during fermentation activates native flour proteases, promoting the breakdown of proteins into peptides and increasing the levels of essential free amino acids, thereby explaining the elevated amino acid content in sourdough and LAB-fermented bread.

## Conclusion

Mixed cultures of *L. plantarum* and *P. pentosaceus* enhanced sourdough bread nutrition by reducing antinutrients, improving metabolizable energy, and enriching amino acids content of the bread. Their synergistic acidification and enzymatic activities position them as ideal starters for nonwheat sourdough systems.

## Data Availability

The data underlying this article are available in the article and in its online supplementary material.
